# Birth preparedness, utilization of skilled birth attendants and delivery outcomes among pregnant women in Ogun State, Nigeria

**DOI:** 10.18332/ejm/120116

**Published:** 2020-05-28

**Authors:** Adekemi E. Olowokere, Adeola T. Oyedele, Abiola O. Komolafe, Aanuoluwapo O. Olajubu

**Affiliations:** 1 Department of Nursing Science, College of Health Sciences, Obafemi Awolowo University, Ile-Ife, Nigeria; 2 Ogun State School of Nursing and Midwifery, Idi-aba, Nigeria

**Keywords:** maternal mortality, birth preparedness and complication readiness, skilled birth attendants, delivery outcomes

## Abstract

**INTRODUCTION:**

Birth preparedness and complication readiness (BPCR) is an approach initiated to facilitate utilization of skill birth attendants (SBAs) for improved pregnancy outcomes. Despite its usefulness, many women still did not use skilled birth attendants. The purpose of this study is to assess the level of birth preparedness and complication readiness and its association with skilled birth attendants’ utilization.

**METHODS:**

A descriptive sequential mixed methods design was used. In all, 350 women in their third trimester were purposively selected from healthcare facilities. Of these, 340 completed the study yielding a 97% response rate. Structured interviewer-administered questionnaire, a checklist and an in-depth interview guide were used to collect data. Data analysis was done in Statistical Package for Social Sciences version 20 using descriptive and inferential statistics at 0.05 level of significance while qualitative data were analyzed through content analysis.

**RESULTS:**

There was a significant association between level of birth preparedness and complication readiness and use of skilled birth attendants [χ^2^(2, 340)=19.96; p=0.0001]. Some negative delivery outcomes (complications) were significantly associated with nonutilization of skill birth attendants. Cost, family members’ preference, distance, industrial action and irritation from the vaginal examination were factors that prevented women from using a skilled birth attendant.

**CONCLUSIONS:**

The study concluded that BPCR is significantly associated with the use of SBAs and better outcomes were observed in women that used SBAs in Nigeria.

## INTRODUCTION

Pregnancy related deaths remain a leading cause of mortality among women of reproductive age. The maternal mortality ratio in Nigeria is still as high as 814 per 100000 live births^1^. Empirical evidence has, however, shown that maternal mortality reduction will be possible if quality obstetric care by a skilled birth attendant is made available^2,3^ and utilized by all pregnant women.

Birth preparedness and complication readiness (BPCR) is a comprehensive approach initiated to facilitate utilization of quality obstetric care that can help improve pregnancy outcomes and reduce maternal mortality^4^. This strategy provides pregnant women with information on susceptibility and severity of danger signs, how to improve accessibility to skilled care and effective interventions in cases of obstetric emergencies^5^. This strategy is needed and is essential in countries with high maternal mortality, such as Nigeria, if Sustainable Development Goal 3 that targets the reduction of world’s maternal mortality ratio to less than 70 maternal deaths per 100000 births (Target 3.1) is to be achieved.

Despite the adoption of BPCR in all the regions, 20% of all global maternal deaths happen in Nigeria^6^. This may not be unconnected with non-utilization of skill birth attendants by many women. Studies have been conducted on BPCR in Nigeria but none of these studies follows up the pregnant women to delivery in order to establish any association between BPCR and use of skilled birth attendants (SBAs) as well as the delivery outcomes among women who utilized SBA and those who did not^7-11^. Also, most of the reported utilizations of skilled birth attendants documented in the literature^7-11^ in Nigeria were based on self-reported intention and previous pregnancy, which are prone to bias and may have compromised the validity of the reports. These gaps were particularly addressed in this study to provide empirical evidence on the association between BPCR and SBA utilization. This is necessary as part of efforts to promote positive pregnancy outcomes and to reduce maternal and neonatal mortality in Nigeria. Skilled birth attendants in this study are healthcare professionals (doctors and nurse-midwives) that are trained and certified by their respective professional council in Nigeria to care for women during pregnancy, childbirth, and the postpartum period.

## METHODS

### Study design

A mixed-method design that was quantitatively-driven (QUAN-QUALI) was used for the study. Data were collected through the use of a structured questionnaire, a checklist and an in-depth interview guide.

### Participants

The target population for the study are pregnant women in Ogun state. The antenatal clinic patronage of healthcare institutions in the past year was 48970^12^. The sample for this study was obtained using a Cochrane formula (n = Z^2^pq/d^2^) for single proportion^13^. The proportion of birth preparedness practice was taken as 71% from a previous study^7^. Substituting the proportion in the formula, a sample size of 316 was obtained, which was increased to 350 to allow for a 10% loss to follow-up. Multistage sampling technique was used to select eight healthcare facilities (one tertiary, four secondary and three primary health facilities) in Ogun Central senatorial district, Ogun state in Southwestern Nigeria. The district was purposively selected among the three senatorial districts in Ogun state because it is the senatorial district where the only tertiary health facility that majors in obstetrics is located. A simple random sampling technique was used to select three Local Government Areas (LGAs) out of the six in the district. Thereafter, one primary healthcare center was randomly selected from each of the three LGAs, and four secondary healthcare facilities that provide maternal and child health services were purposively selected in the three LGAs. Thus, respondents were recruited from 1 tertiary healthcare facility, 4 secondary healthcare facilities and 3 primary healthcare facilities in this study. The sample size calculated was proportionally distributed across the eight facilities based on the monthly antenatal care attendance. Women were purposively selected at facility level and were pregnant women in their third semester. For the qualitative aspect, purposive sampling technique was employed to collect data from a total of 55 women among the study sample who did not come back to the facilities to deliver.

### Data collection procedure

The data were collected from August to December 2016. A pre-tested structured interviewer-administered questionnaire adapted from the safe motherhood tools^5^ was used for the study. An expert translated the English version to the local language (Yoruba), which was back translated into English. The reliability of the questionnaire was established using a test re-test method with a correlation coefficient of 0.85 while the validity was established through face and content validity criteria. The questionnaire was given to experts in maternal and child health nursing for thorough scrutiny. All respondents were first accessed at healthcare facilities to collect data on their sociodemographic characteristics and birth preparedness practices using the structured questionnaire.

A checklist was used to collect data on the use of SBAs and some selected outcomes from the registers and case notes of women who delivered at the healthcare facilities. Thereafter, women who delivered outside healthcare facilities were followed up using their addresses to explore place of delivery, use of SBA, outcomes of delivery and factors responsible for non-utilization of an SBA through an in-depth interview guide. Each in-depth interview lasted 30–45 minutes. Tape-recordings and note taking were used during the interview.

Ethical approval was obtained from Health Research Ethics Committee (HREC) of Federal Medical Centre, Idi Aba, Abeokuta. Official letters were taken from Health Board to the healthcare facilities, and permission was obtained from the heads of the facilities. Written informed consent was obtained from each respondent after explaining the objectives and procedures of the study. The right to participate voluntarily and to withdraw at any time was also made known to the respondents. Issues of confidentiality were maintained by removing any identifiers from the questionnaire. Research assistants were also trained to maintain the confidentiality of information gathered in the course of the study.

### Data analysis

Univariate analysis was done using frequency, percentage, mean, and range, and presented in the form of text, tables, and pictogram. The total obtainable score for practice of BPCR was 10. Scores above first quarter percentiles (7.5), which were scores from 8–10, were considered ‘well prepared’ while scores from 5–7 were categorized as ‘moderately prepared’, and scores below 5 were considered ‘less prepared’. Bivariate analysis was done by using chi-squared test to determine the association between level of BPCR and use of skilled birth attendants. It was also used to determine the association between some selected delivery outcomes and use of a skilled birth attendant, at significance level of p<0.05. The qualitative data were transcribed into English text by the principal investigator and an official bilingual translator by searching for patterns and commonalities among the participants’ responses. Open coding of the collected data was done whereby codes were clustered according to common themes and subcategories. The validity of the data collected from the women was also upheld by asking them to verify the data themselves, vis-à-vis, the issues raised during the discussion. Data were first checked manually for completeness then coded, and entered into Epi-Info version 3.5 statistical software and cleaned thoroughly before being transferred to the Statistical Package for Social Sciences (SPSS) version 20.0 for further analysis.

## RESULTS

### Sociodemographic and obstetric characteristics

The respondents’ sociodemographic and obstetric characteristics are shown in Table 1. The attrition rate in this study was 3% as a total of 340 out of 350 respondents that were recruited into the study remained at follow-up. The age range of respondents was between 14 and 44 years, with a mean age of 29.6 ± 5.3 years. Almost all the respondents (96.8%) were married, and many of them (63.2%) had tertiary education. About two-thirds (61.4%) of women in the study were multigravida while few (29.4%) were primigravida. Also, many of the respondents (65%) attended antenatal care for 4 to 8 times during the present pregnancy.

**Table 1:** Sociodemographic characteristics of the participants (N=340)

*Characteristics*	*n*	*%*
**Age** (years)		
14–20	12	3.5
21–30	191	56.2
31–40	127	37.4
41–50	10	2.9
**Religion**		
Christianity	238	70.0
Islam	99	29.1
Traditional	3	0.9
**Marital status**		
Married	329	96.8
Not married	11	3.2
**Educational level**		
No formal education	6	1.8
Primary	17	5.0
Secondary	102	30.0
Tertiary	215	63.2
**Occupation**		
Artisan	36	10.6
Farmer	5	1.5
Formal employment	94	27.6
Trader	146	42.9
Unemployed	15	4.4
Others	44	12.9
**Gravidity**		
Primigravida	100	29.4
Multigravida	209	61.4
Grand multigravida	31	9.2
**Parity**		
Nullipara	106	31.2
Primipara	81	23.8
2–4 (multipara)	143	42.0
≥5 (grand multipara)	10	3.0
**Antenatal visits**		
1–3	104	30.6
4–8	221	65.0
≥9	15	4.4

### Practice of Birth Preparedness and Complication Readiness

Table 2 shows the BPCR practices of the respondents. The majority (88.2%) of the respondents reported that they were aware of their expected date of delivery. The highest reported birth preparedness and complication readiness practice was the identification of a healthcare facility to deliver (97%) while the least reported practice was making preparation for a blood donor (50.6%).

**Table 2:** Birth preparedness and complication readiness of respondents (N=340)

*BPCR*	*Yes n (%)*	*No n (%)*
I am aware of my expected date of delivery	300 (88.2)	40 (11.8)
I know that labor can start before due date	309 (90.9)	31 (9.1)
I have made funds available for birth and emergency	283 (83.2)	57 (16.8)
I have identified health facility where I will deliver	330 (97.1)	10 (2.9)
I have made preparation for transportation	303 (89.1)	37 (10.9)
I have made preparation for blood donor	172 (50.6)	168 (49.4)
I have already identified the person that will accompany me to the hospital	300 (88.2)	40 (11.8)
I have already identified who will stay with the other children when I leave for hospital	214 (62.9)	126 (37.1)
I have a bag to pack items needed for delivery	318 (93.5)	22 (6.5)
I have packed items needed for delivery	322 (94.7)	18 (5.3)

Table 3 depicts the respondents BPCR indices. The majority of the respondents (83.5%) chose government healthcare facilities as the delivery site while few (13.5%) of the respondents chose private healthcare facilities. The means of transportation chosen by many (66.8%) of the respondents was a private car, and only very few (2.4%) chose public motorcycle. Forty-four per cent (44%) chose their husband as the blood donor while 1.8% chose the blood transfusion service. The results also showed that 49.4% was yet to identify a blood donor. Also, 58.8% chose their husband to accompany them to the healthcare facility. In summary, the majority (76.8%) of the respondents were ‘well prepared’, 20% were ‘moderately prepared’ while few (3.2%) were ‘less prepared’.

**Table 3:** BPCR indices chosen by respondents (N=340)

*BPCR indices*	*n*	*%*
**Delivery site^a^**		
Government	284	83.5
Private	46	13.5
TBAs	2	0.6
Home	4	1.2
Yet to identify delivery site	10	2.9
**Means of transportation**		
Private car	227	66.8
Public car	53	15.6
Private motor cycle	15	4.4
Public motor cycle	8	2.4
Yet to identify transport	37	10.9
**Blood donor**		
Husband	150	44.1
Family	11	3.2
Non-relative	5	1.5
Blood transfusion service	6	1.8
Yet to identify donor	168	49.4
**Person that will accompany to the hospital**		
Husband	200	58.8
Mother	40	11.8
Mother-in-law	31	9.1
One of the children	3	0.9
Friend	13	3.8
Others	13	3.8
Yet to identify the person	40	11.8
**Care of other children**		
Husband	66	19.4
Mother	53	15.6
Mother-in-law	39	11.5
One of the children	10	2.9
Friend	16	4.7
Others	30	8.8
Yet to identify the person	20	5.9
Not applicable (nulliparous)	106	31.2
**Package of delivery of items**		
Complete	198	58.2
Partial	124	36.5
Yet to pack any items	18	5.3

a Multiple response given by some participants.

### Birth Preparedness and Complication Readiness (BPCR) and use of Skilled Birth Attendants (SBAs)

**Figure 1:**
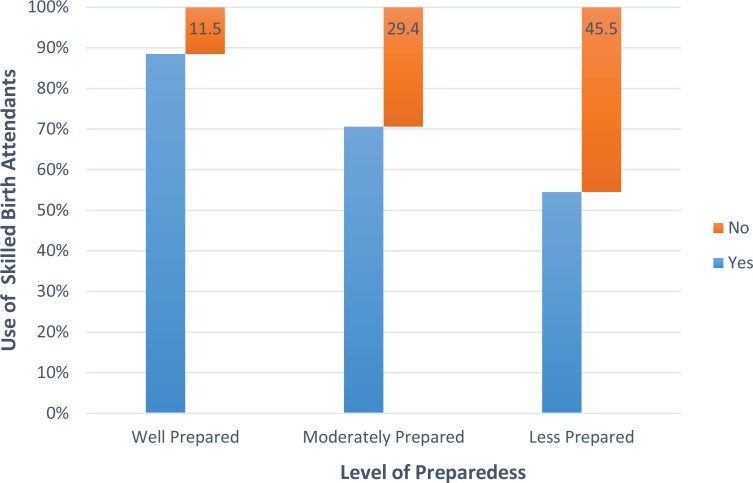
Level of birth preparedness and use of SBAs

The use of SBAs was generally high (83.8%) in this study. Figure 1 shows a bar graph relating the level of birth preparedness with utilization of SBAs. The figure reveals that a higher percentage of women who were well prepared used an SBA. Further analysis using chi-squared test (Table 4) shows a significant association between the level of BPCR and use of skilled birth attendants [χ^2^(2, 340)=19.96; p=0.0001].

**Table 4:** Association between level of BPCR and utilization of SBAs

*Preparedness*	*Utilization of SBAs*		*χ^2^*	*p*
	*Yes n (%)*	*No n (%)*		
Well Prepared	231 (88.5)	30 (11.5)	19.96	0.0001
Moderately Prepared	48 (70.6)	20 (29.4)		
Less Prepared	6 (54.5)	5 (45.5)		

### Use of Skilled Birth Attendants and outcomes of delivery

Table 5 shows the association between some selected outcomes in mothers that used SBAs and those that did not use them. There was a significant association between use of SBAs and being healthy after delivery [χ^2^ (1, 340) = 9.14; p=0.02]. Also, having severe bleeding [χ^2^(1, 340)=4.31; p=0.04] and high-grade fever [χ^2^ (1, 340)=5.45; p=0.02] after delivery were significantly higher among mothers who did not use an SBA.

**Table 5:** Association between selected outcomes (mother) and utilization of SBAs

*Outcomes*	*Utilization of skilled birth attendants*		*χ^2^*	*p*
	*Yes n (%)*	*No n (%)*		
**Healthy mother**				
Yes	268 (94.0)	45 (81.8)	9.142	0.02*
No	17 (6.0)	10 (18.2)		
**Mother experienced severe bleeding after birth**				
Yes	6 (2.1)	4 (7.3)	4.312	0.04*
No	279 (97.9)	51 (92.7)		
**Mother experienced severe headaches**				
Yes	9 (3.2)	2 (3.6)	0.034	0.85
No	276 (96.8)	53 (96.4)		
**Mother experienced convulsions**				
Yes	2 (0.7)	0 (0.0)	0.388	0.53
No	283 (99.3)	55 (100.0)		
**Mother had high grade fever as a result of sepsis**				
Yes	5 (1.8)	4 (7.3)	5.448	0.02*
No	280 (98.2)	51 (92.7)		
**Mother whose placenta was not delivered 30 min after baby**				
Yes	3 (1.1)	2 (3.6)	2.124	0.15
No	282 (98.9)	53 (96.4)		
**Mother experienced severe weakness**				
Yes	8 (2.8)	2 (3.6)	0.111	0.74
No	277 (97.2)	53 (96.4)		

* Statistically significant p<0.05.

Table 6 shows the association between selected outcomes in babies of mothers that used SBAs and those that did not. Having healthy babies was significantly associated with use of SBAs [χ^2^ (1, 340)=4.21; p=0.04]. Also, difficult or fast breathing [χ^2^(1, 340)=5.45; p=0.02] and discharge (pus or bleeding) around the cord [χ^2^ (1, 340)=5.19; p=0.02] were significantly more frequent with babies of mothers who did not use SBAs.

**Table 6:** Association between selected outcomes (baby) and utilization of SBAs

*Outcomes*	*Utilisation of skilled birth attendants*		*X*	*p*
	*Yes (N=285) n (%)*	*No (N=55) n (%)*		
**Healthy baby**				
Yes	252 (88.4)	43 (78.2)	4.21	0.04*
No	33 (11.6)	12 (21.8)		
**Baby had difficult or fast breathing**				
Yes	5 (1.8)	4 (7.3)	5.45	0.02*
No	280 (98.2)	51 (92.7)		
**Baby had yellow skin or eye (jaundice)**				
Yes	18 (6.3)	3 (5.5)	0.06	0.81
No	267 (93.7)	52 (94.5)		
**Baby had poor sucking or feeding**				
Yes	3 (1.1)	1 (1.8)	0.23	0.63
No	282 (98.9)	54 (98.2)		
**Baby had discharge around the umbilical cord**				
Yes	0 (0.0)	1 (1.8)	5.19	0.02*
No	285 (100.0)	54 (98.2)		
**Baby is very small**				
Yes	4 (1.4)	1 (1.8)	0.06	0.82
No	281 (83.9)	54 (98.2)		
**Baby had skin lesions or blisters**				
Yes	2 (0.7)	0 (0.0)	0.39	0.53
No	283 (99.3)	55 (100.0)		
**Baby had convulsions, spasms or rigidity**				
Yes	1 (0.4)	0 (0.0)	0.19	0.66
No	284 (83.8)	55 (100.0)		
**Baby experienced lethargy and unconsciousness**				
Yes	1 (0.4)	0 (0.0)	0.19	0.66
No	284 (83.8)	55 (100.0)		
**Baby had red or swollen eye with pus**				
Yes	1 (0.4)	1 (1.8)	1.69	0.19
No	284 (83.8)	54 (98.2)		
**Still birth**				
Yes	2 (0.7)	2 (3.6)	1.69	0.19
No	283 (99.3)	53 (96.4)		

* Statistically significant at p<0.05.

### Qualitative factors responsible for non-utilization of Skilled Birth Attendants for delivery

The study revealed that none of the women who delivered outside the healthcare facilities received skill birth attendant care. Five major factors emerged as reasons why the women did not use SBAs.

#### Cost of facility-based delivery

An issue relating to cost was one of the reasons why some of the participants did not translate their intention to use SBAs into reality. This could be summarized by some of the participants’ responses:

*‘When I considered the money I had to pay for delivery at the hospital, I knew we would not be able to afford it, so I considered not going to the health facility, believing that God would make everything to be normal.’* (Aged 24 years)

*‘Money caused it; the money I would pay was too much, so I went to Traditional Birth Attendants for delivery.’* (Aged 26 years)

#### Strike action at government hospital

This was mentioned by the respondents as a barrier to translating intention of using skilled birth attendants to actuality. The following represent the expressions of some of the respondents:

*‘I registered at a government hospital, and there was strike action. So, as we were contemplating where to go next, the baby was delivered. That was how my baby was delivered at home.’* (Aged 32 years)

*‘When there was a strike, I decided to use mission home.’* (Aged 27 years)

#### The preference of the family member of the pregnant woman

This also affected some women’s intention of using skilled birth attendants, as expressed below:

*‘I could not deliver on time in the hospital, and my family took me to Traditional Birth Attendant.’* (Aged 25 years)

*‘My husband preferred that I delivered in the Church.’* (Aged 37 years)

#### Distance to a health facility at the onset of labor

This also constituted a barrier to some women:

*‘I travelled to visit my in-law. I could not go to my hospital because it was a little bit far from my in-law place. Somebody was called to assist me. Even though I knew the person was not qualified health personnel, but I had no option.’* (Aged 17 years)

*‘You know my village is quite far from the hospital, and there is often a problem in getting public transport to the place. I miss my antenatal clinic sometimes because of the distance. I delivered my baby at home with the assistance of an elderly woman who works in a mission home and lives in our community.’* (Aged 39 years)

#### Frequency of vaginal examination and associated discomforts

Being irritated by vaginal examination was also mentioned as a barrier to using a skilled birth attendant.

*‘I did not want to go on time so that those nurses would not be doing a vaginal examination on me. I found the frequency of vaginal examination in the hospital very irritating. So, I decided to wait till the labor was strong. I just found that I delivered at home.’* (Aged 38 years)

*‘I had to deliver in the mission home for a singular reason which has to do with the frequency of vaginal examination in the hospital. A doctor will come to examine and do a vaginal examination. Nurses will also come and do a vaginal examination. I do not know maybe they don't trust each other's findings. All of them want to do their own examination. It is very disturbing and painful considering the period when the body has become very sensitive. I wish they could reduce the frequency of the examination.’* (Aged 43 years)

## DISCUSSION

The purpose of this study was to assess the level of birth preparedness and complication readiness (BPCR) and its association with skilled birth attendants’ utilization. The study also determined the association between some selected outcomes of delivery among mothers who used an SBA and those who did not use an SBA, and explored factors that prevented the use of SBAs by some mothers. Overall, the study shows a significant association between BPCR and skilled birth attendants' utilization.

The level of birth preparedness and complication readiness and SBAs' utilization among women in the study setting is high. This is consistent with the findings of studies conducted in the region^10,11^. It was, however, at variance with a study conducted in the northern region of Nigeria^14^ where it was found to be low. Considering the finding of this current study in relation to benefits associated with BPCR and SBAs' utilization, one might be able to explain one of the reasons for high maternal mortality in the northern region compared with other regions in Nigeria. Also, comparing the level of birth preparedness and complication readiness found in this study with other African countries, the result is similar to what was found in a study in Central Tanzania^15^ where most of the study participants were found to be prepared. The result is, however, at variance with studies conducted in Ethiopia^16,17^ and Cameroon^18^ where low BPCR practices were reported.

A possible reason for high level of BPCR in the current study might be that the women were in their third trimester of pregnancy (6–9 months). Women in the third trimester are more likely to have initiated planning activities and be well-prepared compared to those in the first or second trimester^19^. Also, the high level of education attained by many of the respondents is a possible explanation for the high level of BPCR, as was pointed out in previous studies that level of education is a strong predictor of BPCR^20^ and pregnant women that are educated are 6 to 10 times more likely to be prepared for birth and ready for complications than their lower educated counterparts^19,21^. Women with high educational status most likely have socio-economic power to make decisions in matters related to their health and the expected expenses.

The most mentioned practice of birth preparedness and complication readiness in this study is the identification of facility for delivery. This is in agreement with the finding of a previous study carried out in the Southwestern zone of Nigeria^8^. The identification of facility for delivery and the choice of government health facility by many of the respondents may be related to the fact that the two studies were conducted in health facilities. However, studies have documented variation between the choice and the actual use of health facility for delivery among child-bearing women^22,23^. The least mentioned birth preparedness practice is the identification of a blood donor; but the proportion of women who had already identified a donor in this study is lower than what was reported in a previous study in the south region of Nigeria ^11^. Healthcare providers need to continually educate pregnant women on the need to identify a blood donor as part of the birth preparedness and complication readiness practices during antenatal care visits.

There is an association between level of BPCR and use of SBAs. A vast majority of women who were well prepared utilized a skilled birth attendant. This was in line with a previous study that reported that women who were well prepared for childbirth were more likely to choose assistance by an SBA than those who were not well prepared^24^. This, however, runs contrary to the conclusion of a review on skilled birth attendants’ utilization and BPCR, where BPCR does not lead to increased usage of skilled birth attendants^25^. The use of SBAs in women with a high level of BPCR in this study may be due to the fact that the study was carried out in health facilities. A community survey may yield a different result.

The study further revealed that higher proportion of women who did not use SBAs have more obstetric complications. This finding was at variance with the results of some studies in developing countries where obstetric complications were more in women that used skilled providers^26,27^. The major obstetric complications associated with non-utilization of skilled birth attendants were post-partum hemorrhage and high-grade fever as a result of sepsis. Although there was no maternal death in this study, there were more neonatal deaths among babies whose mothers did not utilize an SBA compared with those whose mothers used an SBA. Delivery attendance by an SBA has been reported to be very important in preventing stillbirths and improving new born survival^28^. This study was conducted in the third trimester of pregnancy and this might have sensitized some of the women to utilize an SBA. Nurses need to intensify BPCR and most importantly SBAs' utilization and its benefits as the women progress to the end of pregnancy. This may help them to improve their preparation towards child birth and to take appropriate decisions that can promote positive pregnancy outcomes.

Deductions from the findings of this study revealed that 1 in 6 of the women did not utilized an SBA due to some of the factors reported in the Results section. Low use of SBAs by women has also been reported in previous studies conducted in Ghana^29^ and Nigeria^30^ where cost of skilled care (perceive/real) and poor socioeconomic condition of the women have been found to motivate home, mission and TBA deliveries. To encourage utilization of SBAs, there may be a need for government to subsidize the cost and also initiate a campaign programme from time to time so that the women are aware of the cost and the benefit of using such services.

Another factor, found in this study, that prevents women from using skilled birth attendants is the distance to healthcare facilities. This finding is in support of previous reports in the literature^31,32^. The closeness of women’s residence to healthcare facilities could be a positive influence on the decision to use SBAs. Also, incessant strikes over the years in the public health sector have made many women and their partners lose hope in the system. The private system that they would have considered as an alternative is too expensive for the average family. There is a need for a general overhaul of the healthcare system in Nigeria to address the root cause of issues that often make healthcare workers embark on strike action from time to time.

The preference of a significant family member found in this study to influence utilization of SBAs corroborated previous findings in the literature^33,34^. Most of the time, the focus of healthcare providers on maternity issues is often on women. There is a need to carry health information to the wider society. When the women’s husbands and partners are well informed, they can positively influence their women to utilize SBAs.

Irritation from the vaginal examination was found to be one of the factors why women did not want to use SBAs. This is in support of a previous study^35^ that showed that there is potential for women to perceive vaginal examination as uncomfortable, painful or even abusive. This may hinder many women from patronizing health facilities, most notably when there is no respect for privacy. Nurses and doctors need to work together to avoid repeated vaginal examination on the same woman for the same purpose, especially within a very short interval.

The implication of the factors discussed above is to help healthcare providers plan interventions that can address the barriers to utilization of SBAs in order to encourage utilization of skilled providers.

### Strengths and limitations

The study could not predict a direct causal relationship between BPCR and skilled birth attendant utilization because of the research design. Further work in this area could employ experimental design to establish whether there is a direct causal relationship between BPCR, SBAs utilization and the selected delivery outcomes. Also, this study is limited by being a facility-based study. A community-based study might have yielded a different result. This study needs to be interpreted in light of these limitations. The major strength of this study is the follow up of women from pregnancy to birth in order to assess the instant situation or fresh memory of the mothers, thereby preventing recall bias. The mixed-methods approach employed by this study offers the additional benefit of a qualitative method to explore the actual reasons why women did not utilize skilled birth attendant care.

## CONCLUSIONS

The study shows that birth preparedness and complication readiness (BPCR) is significantly associated with the use of a skilled birth attendant, and better outcomes were observed in women that used skilled birth attendants. Therefore, healthcare professionals should intensify efforts to promote birth preparedness and complication readiness to further improve utilization of skilled birth attendants.
